# Vaccination and food consumption: association with Post-Acute COVID-19 Syndrome in Brazilian adults (CUME Study)

**DOI:** 10.3389/fnut.2025.1549747

**Published:** 2025-03-14

**Authors:** Marlise Lima Brandão, Helen Hermana Miranda Hermsdorff, Arieta Carla Gualandi Leal, Josefina Bressan, Adriano Marçal Pimenta

**Affiliations:** ^1^Posgraduate Program in Nursing, Universidade Federal do Paraná, Curitiba, Paraná, Brazil; ^2^Department of Nutrition and Health, Universidade Federal de Viçosa, Viçosa, Minas Gerais, Brazil; ^3^Department of Nursing, Universidade Federal do Paraná, Curitiba, Paraná, Brazil

**Keywords:** Post-Acute COVID-19 Syndrome, COVID-19, eating, vaccination, cohort studies

## Abstract

**Background:**

Post-Acute COVID-19 Syndrome (PACS) is an important sequalae of COVID-19. Then, our objective was to analyze the risk and protective factors for PACS in Brazilian adults participating in the Cohort of Universities of Minas Gerais (CUME Study), with emphasis on COVID-19 vaccination and food consumption.

**Methods:**

In this sub-study, we included 2,065 participants of CUME Study who answered the baseline questionnaire in 2016 or 2018 or 2020 or 2022, and the follow-up COVID-19/PACS-specific questionnaire in 2023. PACS diagnosis was based on self-reporting of continuation or development of new symptoms 3 months after the initial SARS-CoV-2 infection, with these symptoms lasting for at least 2 months with no other explanation. To estimate the risk and protective factors for PACS, hierarchical multivariate statistical analysis was conducted using the Cox regression technique, producing two models: (1) focusing on consumption of macro and micronutrients; (2) focusing on consumption of food groups.

**Results:**

After a median of 5.5 years of follow-up, 54.4% of the participants reported PACS. When we analyzed the consumption of macro and micronutrients, higher intake of proteins (HR: 1.36; 95% CI: 1.06–1.74-4th quartile) and lipids (HR: 1.23; 95% CI: 1.02–1.48-4th quartile) were risk factors for PACS. On the other hand, higher intake of vitamin C (HR: 0.78; 95% CI: 0.64–0.94-4th quartile), vitamin D (HR: 0.81; 95% CI: 0.67–0.99-4th quartile), and zinc (HR: 0.66; 95% CI: 0.52–0.83-4th quartile) were protective factors for the outcome (model 1). When we analyzed the consumption of food groups, higher intake of eggs (HR: 1.59; 95% CI: 1.34–1.89-4th quartile) increased the risk of PACS, whereas, respectively, higher and intermediate consumption of white meat (HR: 0.84; 95% CI: 0.71–1.00-4th quartile) and vegetables (HR: 0.81; 95% CI: 0.67–0.99-2nd quartile; HR: 0.81; 95% CI: 0.67–0.99-3rd quartile) decreased the risk of the outcome (model 2). In both models, pre-infection COVID-19 vaccination was a protective factor for PACS.

**Conclusion:**

A healthy diet, with higher consumption of white meat, vegetables and specific micronutrients (vitamin C, vitamin D, zinc), in parallel with pre-infection COVID-19 vaccination, is essential to reduce the risk of PACS.

## Introduction

COVID-19 was declared a public health emergency of international concern between January 30, 2020 and May 5, 2023. During this period, the disease was responsible for over 770 million cases and 7 million deaths worldwide (1, 2).

Currently, we are experiencing an endemic period of COVID-19. However, the disease continues to pose a significant global health challenge, as individuals still experience illness, death, and sequelae stemming from its acute phase (3).

Among these sequelae, Post-Acute COVID-19 Syndrome (PACS) is particularly notable. The World Health Organization (WHO) defines PACS as “the continuation or development of new symptoms of COVID-19 3 months after the initial SARS-CoV-2 infection, with these symptoms lasting for at least 2 months with no other explanation” (3).

A meta-analysis estimated the average prevalence of PACS to be 64% (4). This high prevalence has become a substantial challenge for healthcare professionals and systems worldwide. Understanding the risk and protective factors for PACS is therefore essential for the development of effective strategies, policies, and health programs aimed at its prevention.

Scientific findings consistently identified the following risk factors: (a) socioeconomic, such as female sex, advancing age, white skin color, and low income (5–12); (b) health conditions, such as pre-existing diagnoses of obesity (8, 9, 13), hypertension (14, 15), diabetes (16), dyslipidemia (8, 17), and chronic respiratory diseases (12, 18, 19); (c) clinical conditions related to the acute phase of COVID-19, such as the number of symptoms (5–7, 14, 20, 21), severity (5, 22–24), and hospitalization (12, 14, 16, 25). Conversely, protective factors have been identified, particularly infection with the Omicron variant (5, 6) and being infected during later waves of the acute COVID-19 phase (26).

Despite these findings, few studies have explored the relationship between the status of COVID-19 vaccination (pre- or post-infection) and the occurrence of PACS (27, 28), leaving this area of scientific evidence underdeveloped. Additionally, to our knowledge, no studies have investigated how macronutrient and micronutrient intake or the specific consumption of food groups might influence the risk of PACS incidence.

Therefore, the objective of this study was to analyze the risk and protective factors for PACS in Brazilian adults participating in the Cohort of Universities of Minas Gerais (CUME Study), with an emphasis on COVID-19 vaccination and food consumption.

## Materials and methods

### The CUME Study

The CUME Study is an open prospective cohort initiated in 2016. The cohort includes graduates of both sexes, aged 18 years or older, from seven public federal higher education institutions located in the state of Minas Gerais, Brazil. Its primary objective is to analyze the impact of the dietary patterns of the Brazilian population and nutritional transition on non-communicable chronic diseases.

Participant recruitment is ongoing, allowing for continuous sample growth with each follow-up wave, which occurs every 2 years. Previously recruited participants complete follow-up questionnaires (Q_2, Q_4, Q_6…, Q_n), while new participants respond to the baseline questionnaire (Q_0).

Further details regarding the study design, dissemination strategies, and the profile of the initial baseline participants are available in a prior publication (30).

As the CUME Study is a multicenter study, there may be similarities in the processes of variable definition, description, categorization, and data collection across publications derived from its database. For example, we have recently published the findings of the pilot study of the present research (31).

### Ethical considerations

The CUME Study was conducted in accordance with the guidelines established in the Declaration of Helsinki. All procedures involving study participants were reviewed and approved by the Research Ethics Committee of the participating institutions (CAAE: 67808923.7.1001.5153). An informed consent form was obtained from all participants prior to their inclusion in the study.

### Data collection

Data collection is conducted through a virtual platform where participants access the informed consent form, and the questionnaires. After agreeing to the informed consent form, participants are directed to complete the respective questionnaires.

The present research is a sub-study that included participants who answered the baseline questionnaire (Q_0) in March–August 2016 or March–August 2018 or March–August 2020 or March–September 2022, and the follow-up COVID-19/PACS-specific questionnaire (Q_CoV) in October–November 2023.

The baseline questionnaire of the CUME Study (Q_0) was structured into two sections: the first contemplated socioeconomic variables, lifestyle habits, self-reported morbidities, continuous medication use, clinical and laboratory exams conducted within the past 2 years, and anthropometric data. The second was titled Food Frequency Questionnaire (FFQ) which was comprised of 144 items, categorized into eight food groups (dairy products, meats and fish, cereals and legumes, oils and fats, fruits and vegetables, beverages, and other foods or prepared dishes).

The COVID-19/PACS-specific questionnaire (Q_CoV) is the main data collection instrument of this sub-study. It contained questions about COVID-19 occurrence, testing, symptoms, hospitalization, vaccination, PACS occurrence, and PACS symptoms.

### Sample, inclusion, and exclusion criteria

All participants who completed the baseline (Q_0) and the COVID-19/PACS-specific (Q_CoV) questionnaires were included (*n* = 3,666). The following exclusion criteria were applied: (a) Participants without a prior diagnosis of COVID-19 (*n* = 1,298), as only individuals who had experienced the acute phase of the disease could develop PACS; (b) Foreign participants (*n* = 11), as cultural differences might influence both the exposure and the outcome variables; (c) Brazilian participants living abroad (*n* = 253), as different countries adopted varying measures to combat the COVID-19 pandemic and have diverse healthcare system structures, potentially affecting both the exposure and outcome variables; (d) Participants reporting extreme energy intakes [(≤500 kcal/day or ≥6,000 kcal/day), *n* = 39], which could indicate low accuracy in dietary intake data (29).

**Figure 1:**
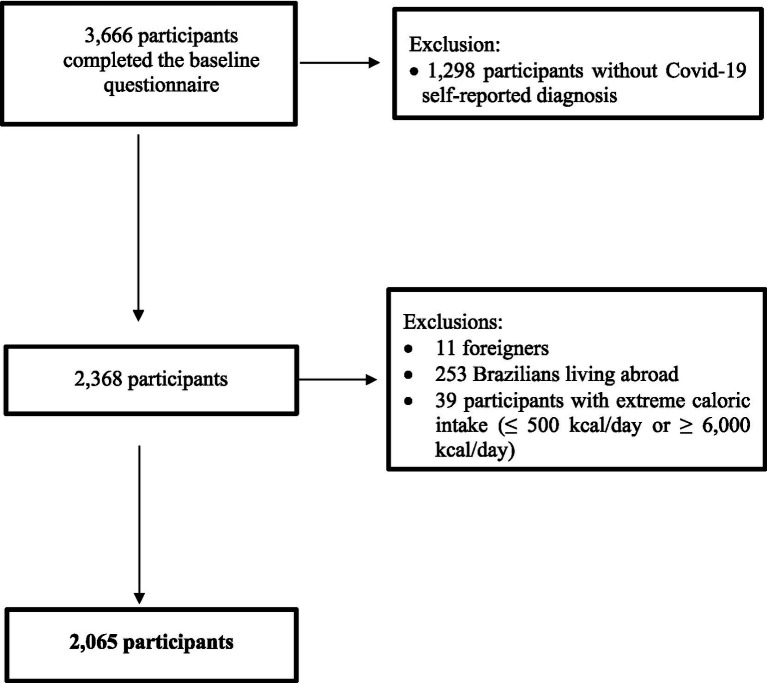
Flowchart of participant inclusion in the CUME Study, 2016–2023.

After applying these exclusion criteria, the final sample for this study consisted of 2,065 participants (Figure 1).

### Outcome variable: Post-Acute COVID-19 Syndrome

The COVID-19/PACS-specific questionnaire (Q_CoV) included a question to assess PACS, based on the World Health Organization (WHO) definition: “PACS is defined as the continuation or development of new COVID-19 symptoms 3 months after the initial SARS-CoV-2 infection, with these symptoms lasting for at least 2 months with no other explanation” (3). Participants were asked to check the main symptoms they currently experienced or had experienced, considering this definition: intense fatigue; chronic pain; liver diseases; muscle weakness; difficulty breathing; cognitive deficits, such as changes in memory; neurological symptoms, such as loss of smell, dizziness, and headaches; anxiety disorders, and post-traumatic stress.

Participants were classified as not having PACS if they reported no symptoms, while those reporting one or more symptoms were classified as having PACS.

### Exposure variables: risk and protective factors for Post-Acute COVID-19 Syndrome

Exposure variables were obtained from the baseline questionnaire (Q_0) and categorized according to CUME Study standards, as described in previous studies (30, 31), namely: (a) Socioeconomic: biological sex (female, male), age group (20–29 years old, 30–39 years old, 40–49 years old, 50–74 years old), skin color (white–yes and black/brown/yellow/indigenous–no), marital status (in a stable union–yes and single/widowed/separated/divorced–no), area of study (health sciences–yes and earth and exact sciences/biological sciences/engineering/applied social sciences/agricultural sciences/linguistics/study of languages and arts–no), educational level (graduation/specialization, master’s, doctorate/post-doctorate), professional situation (working–yes and retired, student, unemployed–no), family income (<4 minimum wages; 5–9 minimum wages, ≥10 minimum wages); (b) Lifestyle variables included current smoking (yes and no), heavy episodic drinking (yes and no), physical activity (sedentary, insufficiently active, active), dietary intake (food groups: dairy, red meat, white meat, eggs, cereals and legumes, fats and oils, fruits, vegetables, alcohol; macronutrients: carbohydrates, proteins and lipids; micronutrients: iron, vitamin C, vitamin D, magnesium, selenium, zinc); (c) Health conditions: self-reported medical diagnoses of obesity (yes and no), hypertension (yes and no), type 2 diabetes mellitus (yes and no), dyslipidemia (yes and no), hypercholesterolemia (yes and no), hypertriglyceridemia (yes and no), high levels of LDL-c (yes and no), low levels of HDL-c (yes and no), asthma (yes and no) and bronchitis (yes and no).

Additionally, some covariates were obtained from the COVID-19/PACS-specific questionnaire (Q_CoV), namely: number of times infected with SARS-CoV-2 (1, 2, 3, 4 and ≥5), waves of infection (first, second, third, endemic period, post-pandemic period), number of COVID-19 symptoms (0, 1–2, 3–5 and ≥6), seeking healthcare (yes and no), hospitalization (yes and no), vaccination status (received the vaccine before COVID-19 infection, received the vaccine after COVID-19 infection).

Family income was categorized in minimum wages, according to the value in force in the year of data collection.

Heavy episodic drinking was defined as the consumption of four or more drinks of any alcoholic beverage for women and five or more drinks of any alcoholic beverage for men on a single occasion within the past 30 days (32).

Physical activity was assessed using a list of 24 leisure activities, described in minutes per week. Initially, it was categorized as light, moderate and vigorous, and then the variable “level of physical activity” was created, categorized as “active” (≥150 min/week of moderate intensity or ≥75 min/week of vigorous activity or ≥150 min/week of vigorous and moderate intensity); insufficiently active” (<150 min/week of moderate intensity; <75 min/week of vigorous intensity; <150 min/week of vigorous and moderate intensity; light intensity activities); and inactive (absence of physical activity during leisure time) (33).

The FFQ filled out by each participant provided information on their dietary intake. Participants selected the items from the food groups they consumed during the year prior to study and, when selecting a food, had to describe the portion sizes consumed in household measures (teaspoon; tablespoon; ladle; knife tip; pasta server; saucer; cup or glass) or traditional portions (units; slices; or pieces). Subsequently, the frequencies of weekly, monthly and annual intake of each food were transformed into daily consumption. Then, the daily food intake, in grams or milliliters, was calculated (portion size versus consumption frequency). The values of energy intake (Kcal) and nutrients were calculated according to data provided in the Table of Reference Measures for Foods Consumed in Brazil (34), using as a complement the Brazilian Food Composition Table (35) and data from the United States Department of Agriculture (36).

Dietary intakes according to food groups, macronutrients and micronutrients were adjusted for caloric intake using the residual method (37) and were previously validated in a sub-sample of the CUME Study (38).

The percentage contribution of daily energetic intake (%/d) of macronutrients was obtained by dividing the total consumption of each macronutrient by total energy intake, and classified according to dietary reference intakes: Carbohydrates: adequate = 45–65%, low <45%, high ≥65%; Proteins: adequate = 10–35%, low <10%, high ≥35%; Lipids: adequate = 25–35%, low <25%, high ≥35% (39).

We also classified the consumption of micronutrients according to dietary reference intakes: Iron: adequate (men = 6–45 mg/d; women aged 19–50 = 8.1–45 mg/d; women aged ≥50 = 5–45 mg/d), low (men <6 mg/d; women aged 19–50 <8.1 mg/d; women aged ≥50 <5 mg/d), high ≥45 mg/d; Vitamin C: adequate (men = 75–2,000 mg/d; women = 60–2,000 mg/d), low (men <75 mg/d; women <60 mg/d), high ≥2,000 mg/d; Vitamin D: adequate (people aged 19–50 = 5–50 μg/d; people aged 51–70 = 10–50 μg/d; people aged ≥70 = 15–50 μg/d), low (people aged 19–50 <5 μg/d; people aged 51–70 <10 μg/d; people aged ≥70 <15 μg/d), high ≥50 μg/d; Magnesium: adequate (men aged 19–30 = 330–350 mg/d; men of other ages = 350 mg/d; women aged 19–30 = 255–350 mg/d; women of other ages = 265–350 mg/d); high ≥350 mg/d; Selenium: adequate = 45–400 μg/d, low <45 μg/d, high ≥400 μg/d; Zinc: adequate (men = 9.4–40 mg/d; women = 6.8–40 mg/d), low (men <9.4 mg/d; women <6.8 mg/d), high ≥40 mg/d (39).

Obesity was defined according to the cut-off point proposed by WHO (Body Mass Index–BMI ≥ 30 kg/m^2^) (40). Hypertension was considered when the participants self-reported a medical diagnosis of the disease or systolic blood pressure ≥ 140 mmHg or diastolic blood pressure ≥ 90 mmHg or use of antihypertensive medication (41). Type 2 diabetes mellitus was also considered when the participants self-reported a medical diagnosis of the disease or glycemia ≥126 mg/dL or using oral antidiabetic medication or using insulin (42). Hypercholesterolemia, hypertriglyceridemia, high blood levels of LDL-c and low blood levels of HDL-c were identified when participants self-reported, respectively, cholesterol ≥190 mg/dL, triglycerides ≥150 mg/dL, LDL-c ≥ 130 mg/dL and HDL-c < 40 mg/dL (43). Finally, if the participants had hypercholesterolemia and/or hypertriglyceridemia and/or high blood levels of LDL-c and/or low blood levels of HDL-c, they were classified with dyslipidemia (43).

In a previous study conducted with a sub-sample of the CUME Study, the data of weight, height, BMI, cholesterol, triglycerides, HDL-c, glycemia and blood pressure, presented moderate to excellent agreement between self-reports and measurements taken directly by the researchers. Moreover, the medical diagnosis of hypertension and the medical diagnosis of type 2 diabetes mellitus were also validated (44).

In Brazil, three waves of the pandemic period of COVID-19 have been described: the first from February 23, 2020 to November 7, 2020; the second from November 8, 2020 to December 25, 2021; and the third from December 26, 2021 to May 21, 2022 (45). Additionally, we also included two other periods of COVID-19 occurrence in the present study: endemic (between May 22, 2022 and May 4, 2023) and post-pandemic (between May 5, 2023 and the date of the last day of data collection of the current study, November 30, 2023) (46).

The status of COVID-19 vaccination (pre-or post-infection) was based on the participants’ responses to the dates of the first SARS-CoV-2 infection and the first dose of immunization.

### Data analysis

Initially, participants were characterized by presenting absolute and relative frequencies, means, standard deviation, median, and interquartile range (IQR) of their socioeconomic variables, lifestyle habits, food consumption, self-reported health conditions, COVID-19 clinical conditions, and COVID-19 vaccination status, stratified by the occurrence or non-occurrence of PACS.

Statistical differences were evaluated using Pearson’s chi-squared and the Mann–Whitney tests. A hierarchical multivariate analysis was conducted using the Cox regression technique, which is used to obtain estimates of the proportional Hazard Ratio (HR) (47), where the outcome variable becomes the time until the event occurs, and participants are counted as person-time (47).

Thus, the time to the occurrence of PACS was calculated in person-years for each participant as follows: (1) difference between the date of self-reported PACS and the date of completion of the baseline questionnaire (Q_0); (2) difference between the date of completion of the COVID-19/PACS-specific questionnaire (Q_CoV) and the date of completion of the baseline questionnaire (Q_0) when PACS was not self-reported.

To estimate the independent risk and protective factors for PACS, we fitted two models, dividing the variables into four blocks: (1) distal block = socioeconomic; (2) intermediate block 1 = lifestyle habits and food consumption; (3) intermediate block 2 = self-reported health conditions; (4) proximal block = COVID-19 clinical conditions and vaccination status against COVID-19. The differences between the two models were in intermediate block 1. The focus of model 1 was the consumption of macro and micronutrients, whereas in model 2, the focus was the consumption of food groups.

During the first stage, variables associated with PACS at a statistical significance level of 20% in the bivariate analysis were selected for the final model. Then, each variable in the distal block was inserted into the final model in descending order of statistical significance and removed one by one using the backward method until only those with statistical significance levels below 5% remained. Next, the same process was done for the variables in the other blocks. Therefore, in the end, the variables from the previous block adjusted the variables from the subsequent block.

## Results

### Frequencies of Post-Acute COVID-19 Syndrome and its symptoms

The current study included a total of 2,065 participants (571 males and 1,494 females). After a median of 5.5 years of follow-up (approximately 10,752 person-years), 1,124 participants reported symptoms of PACS (54.4%), in the following order of magnitude: cognitive deficits, such as changes in memory (74.2%); intense fatigue (43.0%); neurological symptoms, such as loss of smell, dizziness, and headaches (32.5%); muscle weakness (27.7%); anxiety disorders and post-traumatic stress (22.3%); difficulty breathing (15.2%); chronic pain (9.9%); and liver diseases (1.1%).

### Descriptive analysis

Participants with PACS were more likely to be female and older, have a graduate/specialization level of education, and lower family income (Table 1). Moreover, the consumption of vegetables and zinc were, respectively, higher and lower among participants with the outcome (Table 2).

**Table 1:** Socioeconomic, lifestyle and food consumption characteristics of participants according to the diagnosis of Post-Acute COVID-19 Syndrome.

Characteristics	Post-Acute COVID-19 Syndrome		Total (*n* = 2,065)
	No (*n* = 941)	Yes (*n* = 1,124)	
Socioeconomic (%)			
Female sex*	65.9	77.8	72.4
Age (years)*			
20–29	25.5	27.5	26.6
30–39	46.8	41.0	43.6
40–49	15.4	21.1	18.5
50–74	12.3	10.4	11.3
White skin color	66.5	65.8	66.1
Without stable union	50.1	45.9	47.8
Non healthcare professional	70.8	72.7	71.8
Level of education*			
Bachelor/Specialization’s degree	44.3	52.5	48.8
Master’s degree	32.1	28.7	30.2
Doctoral/Postdoctoral’s degree	23.6	18.9	21.0
Working	77.6	76.3	76.9
Family income (minimum monthly salaries)*			
<4	20.5	30.8	26.1
5–9	34.9	31.1	32.8
≥10	44.6	38.1	41.1
Lifestyle (%)			
Smoking	7.7	8.1	7.9
Binge drinking	41.1	37.4	39.1
Physical activity			
Sedentary	19.9	25.4	22.9
Insufficient	22.3	22.2	22.3
Active	57.8	52.4	54.9

CUME Study, 2016–2023.

*p-value < 0.05 by Pearson’s chi-square.

**Table 2:** Food consumption characteristics of participants according to the diagnosis of Post-Acute COVID-19 Syndrome.

Food consumption characteristics	Post-Acute COVID-19 Syndrome											
	No (*n* = 941)				Yes (*n* = 1,124)				Total (*n* = 2,065)			
	Mean	SD	Median	IQR	Mean	SD	Median	IQR	Mean	SD	Median	IQR
Food groups (grams/day)												
Dairy	237.1	193.8	201.6	97.9–331.9	223.8	182.9	192.4	96.1–302.1	229.9	188.0	195.3	97.2–317.1
Red meat	107.8	83.3	99.9	62.1–139.6	105.2	86.2	96.8	57.7–137.0	106.4	84.9	98.8	60.5–138.9
White meat	93.1	85.5	79.8	48.0–112.9	95.2	96.7	79.4	46.8–119.0	94.2	91.8	79.7	47.6–116.6
Eggs	25.0	36.1	12.1	5.5–30.0	26.4	37.6	13.6	6.0–34.8	25.8	37.0	12.9	5.8–33.0
Cereals	227.2	119.6	215.1	163.1–277.2	225.8	118.3	216.3	161.5–277.1	226.4	118.9	215.9	162.1–277.1
Legumes	87.5	106.9	68.7	35.3–110.4	85.6	110.4	64.3	32.6–106.9	86.5	108.8	67.4	33.8–108.84
Fats and oils	19.2	16.0	16.5	10.1–25.3	20.3	15.7	16.7	10.3–27.1	19.8	15.8	16.6	10.2–26.4
Fruits	449.9	337.2	407.2	253.8–594.1	449.2	331.2	400.8	256.6–581.7	449.5	333.9	402.1	254.9–588.9
Vegetables*	212.4	129.1	192.1	137.2–261.7	230.2	147.4	198.5	144.2–285.2	222.1	139.6	195.3	141.3–271.5
Alcohol	6.1	8.9	3.4	1.2–8.5	5.6	8.9	3.0	1.0–7.8	5.8	8.9	3.2	1.1–8.1
Macronutrients (% energy)												
Carbohydrates	43.9	9.0	44.1	38.2–49.5	44.0	9.1	44.0	38.6–50.2	44.0	9.1	44.1	38.5–49.8
Proteins	17.8	4.3	17.6	15.1–20.0	17.8	4.5	17.3	14.8–20.1	17.8	4.4	17.4	14.9–20.0
Lipids	36.4	7.2	36.1	31.7–40.8	36.5	7.1	36.3	31.9–40.7	36.4	7.2	36.2	31.8–40.8
Micronutrients (milligrams/day)												
Iron	12.5	2.9	12.1	11.0–13.5	12.4	2.9	12.2	11.0–13.4	12.4	2.9	12.1	11.0–13.4
Vitamin C	251.1	224.9	209.4	142.4–311.3	251.8	218.4	216.1	143.7–304.4	251.5	221.4	212.3	142.9–308.9
Vitamin D^†^									4.2	3.0	3.6	2.5–5.1
Magnesium	377.5	86.1	369.7	325.9–424.1	373.4	88.1	363.7	320.9–416.1	375.3	87.2	366.0	322.4–419.8
Selenium^†^	190.4	116.5	163.6	132.7–214.8	188.1	115.7	166.2	125.9–212.8	189.1	116.0	164.9	129.8–214.6
Zinc*	12.9	3.1	12.8	11.3–14.3	12.7	3.1	12.6	11.0–14.1	12.8	3.1	12.7	11.1–14.2

CUME Study, 2016–2023.

IQR = interquartile range; *p-value < 0.05 by Mann–Whitney test; ^†^micrograms/day. SD = standard deviation.

Most participants had adequate consumption of proteins, iron, selenium and zinc. On the other hand, most participants had low consumption of carbohydrates and vitamin D, and high consumption of lipids and magnesium. Also, the intake of this last micronutrient was lower among participants with PACS (Supplementary Table 1).

Participants with PACS also were more likely to have prior diagnoses of obesity, hypertension, hypertriglyceridemia, and bronchitis. They also contracted COVID-19 twice or more times, were infected in the second wave of the pandemic period, had six or more COVID-19 symptoms, sought healthcare, were hospitalized, and received the vaccine after COVID-19 infection (Table 3).

**Table 3:** Health and COVID-19 clinical conditions of participants according to the diagnosis of Post-Acute COVID-19 Syndrome.

Characteristics	Post-Acute COVID-19 Syndrome		Total (*n* = 2,065)
	No (*n* = 941)	Yes (*n* = 1,124)	
Self-reported health conditions (%)			
Obesity*	10.5	16.6	13.8
Hypertension*	10.1	13.2	11.8
Diabetes *mellitus* type 2	3.0	3.2	3.1
Dyslipidemias	43.9	46.6	45.4
Hypercholesterolemia	18.0	17.4	17.7
Hypertriglyceridemia*	8.9	12.5	10.9
High blood levels of LDL-c	11.8	11.3	11.5
Low blood levels of HDL-c	22.3	22.8	22.6
Asthma	5.6	7.7	6.7
Bronchitis*	4.8	7.3	6.2
COVID-19 clinical conditions (%)			
Number of infections*			
1	76.5	62.7	69.0
2	19.3	29.8	25.0
3	3.7	6.3	5.1
4	0.4	0.7	0.6
≥5	0.0	0.4	0.2
Waves of the infection*			
First	5.3	8.0	6.8
Second	27.9	37.6	33.2
Third	26.8	26.6	26.7
Endemic period	35.7	24.6	29.7
Post pandemic period	4.3	3.2	3.7
Number of symptoms*			
0	11.0	3.4	6.8
1–2	24.6	11.4	17.4
3–5	45.4	39.6	42.2
≥6	19.1	45.6	33.6
Searched for health care*	50.9	70.6	61.3
Hospitalization (*n* = 1,273)*	0.8	2.9	2.1
Vaccinal status*			
Post-infection COVID-19	18.2	32.7	26.1
Pre-infection COVID-19	81.8	67.3	73.9

CUME Study, 2016–2023.

*p-value < 0.05 by Pearson’s chi-square test.

### Risk and protective factors for Post-Acute COVID-19 Syndrome

The hierarchical multivariate model of risk and protective factors for PACS, focusing on macro and micronutrients consumption (model 1) is presented in Table 4. Independent risk factors for PACS were: female (HR: 1.30; 95% CI: 1.13–1.49); age between 40 and 49 (HR: 1.40; 95% CI: 1.17–1.68); higher consumption of proteins (HR: 1.36; 95% CI: 1.06–1.74-4th quartile) and lipids (HR: 1.23; 95% CI: 1.03–1.47-3rd quartile; HR: 1.23; 95% CI: 1.02–1.48-4th quartile); prior diagnoses of obesity (HR: 1.45; 95% CI: 1.23–1.71), hypertriglyceridemia (HR: 1.24; 95% CI: 1.03–1.49), and bronchitis (HR: 1.26; 95% CI: 1.00–1.58); the number of COVID-19 infections (HR: 1.14; 95% CI: 1.04–1.25); having contracted COVID-19 in the second pandemic wave (HR = 1.56; 95% CI = 1.13–2.16); the number of COVID-19 symptoms (HR: 1.11; 95% CI: 1.08–1.13); and having sought healthcare during the acute phase of COVID-19 (HR: 1.33; 95% CI: 1.15–1.54). On the other hand, the independent protective factors for PACS were: higher family income (HR: 0.61; 95% CI: 0.52–0.71-5–9 minimum monthly salaries; HR: 0.51; 95% CI: 0.44–0.59-≥10 minimum monthly salaries); higher consumption of vitamin C (HR: 0.78; 95% CI: 0.64–0.94-4th quartile), vitamin D (HR: 0.81; 95% CI: 0.67–0.99-4th quartile), and zinc (HR: 0.78; 95% CI: 0.64–0.97-3rd quartile; HR: 0.66; 95% CI: 0.52–0.83-4th quartile); and pre-infection COVID-19 vaccination (HR: 0.69; 95% CI: 0.56–0.85).

**Table 4:** Hierarchical multivariate model of risk and protective factors for Post-Acute COVID-19 Syndrome, highlighting macro and micronutrients.

Characteristics	Post-Acute COVID-19 Syndrome		*p*-value*
	HR	95% CI	
Distal block			
Sex			
Male	1 (Ref.)	–	–
Female	**1.30**	**1.13–1.49**	**<0.001**
Age (years)			
20–29	1 (Ref.)	–	–
30–39	1.00	0.86–1.16	0.975
40–49	**1.40**	**1.17–1.68**	**<0.001**
50–74	1.17	0.94–1.46	0.153
*p* for trend	–	–	**0.002**
Family income (minimum monthly salaries)			
<4	1 (Ref.)	–	–
5–9	0.61	0.52–0.71	**<0.001**
≥10	0.51	0.44–0.59	**<0.001**
*p* for trend	–	–	**<0.001**
Intermediate block 1			
Macronutrients			
Proteins (% energy)			
1st quartile	1 (Ref.)	–	–
2nd quartile	1.05	0.86–1.28	0.664
3rd quartile	1.02	0.81–1.29	0.841
4th quartile	**1.36**	**1.06–1.74**	**0.016**
*p* for trend	–	–	**0.011**
Lipids (% energy)			
1st quartile	1 (Ref.)	–	–
2nd quartile	0.96	0.81–1.14	0.661
3rd quartile	**1.23**	**1.03–1.47**	**0.021**
4th quartile	**1.23**	**1.02–1.48**	**0.027**
*p* for trend	–	–	**0.007**
Micronutrients			
Vitamin C (mg/d)			
1st quartile	1 (Ref.)	–	–
2nd quartile	0.87	0.73–1.03	0.109
3rd quartile	0.93	0.77–1.11	0.414
4th quartile	**0.78**	**0.64–0.94**	**0.010**
*p* for trend	–	–	**0.018**
Vitamin D (μg/d)			
1st quartile	1 (Ref.)	–	–
2nd quartile	0.85	0.72–1.02	0.082
3rd quartile	0.83	0.69–1.00	0.056
4th quartile	**0.81**	**0.67–0.99**	**0.035**
*p* for trend	–	–	0.076
Zinc (mg/d)			
1st quartile	1 (Ref.)	–	–
2nd quartile	0.87	0.72–1.04	0.130
3rd quartile	**0.78**	**0.64–0.97**	**0.024**
4th quartile	**0.66**	**0.52–0.83**	**0.001**
*p* for trend	–	–	**<0.001**
Intermediate block 2			
Obesity			
No	1 (Ref.)	–	–
Yes	**1.45**	**1.23–1.71**	**<0.001**
Hypertriglyceridemia			
No	1 (Ref.)	–	–
Yes	**1.24**	**1.03–1.49**	**0.022**
Bronchitis			
No	1 (Ref.)	–	–
Yes	**1.26**	**1.00–1.58**	**0.045**
Proximal block			
Number of COVID-19 infections (continuous)	**1.14**	**1.04–1.25**	**0.007**
Waves of COVID-19 infection			
First	1 (Ref.)	–	–
Second	1.21	0.94–1.55	0.134
Third	**1.56**	**1.13–2.16**	**0.008**
Endemic period	1.20	0.86–1.66	0.286
Post pandemic period	1.56	0.98–2.46	0.059
Number of COVID-19 Symptoms (continuous)	**1.11**	**1.08–1.13**	**<0.001**
Searched for health care			
No	1 (Ref.)	–	–
Yes	**1.33**	**1.15–1.54**	**<0.001**
Vaccinal status*			
After COVID-19 infection	1 (Ref.)	–	–
Before COVID-19 infection	**0.69**	**0.56–0.85**	**<0.001**

CUME Study, 2016–2023.

HR = Hazard Ratio; 95% CI = 95% Confidence Interval; *p-value from Cox regression; Proteins: 1st quartile = 6.2–14.9%, 2nd quartile = 15.0–17.3%, 3rd quartile = 17.4–19.9%, 4th quartile = 20.0–46.0%; Lipids: 1st quartile = 11.4–31.7%, 2nd quartile = 31.8–36.1%, 3rd quartile = 36.2–40.7%, 4th quartile = 40.8–77.8%; Vitamin C: 1st quartile = 0.0–142.8 mg/d, 2nd quartile = 142.9–212.2 mg/d, 3rd quartile = 212.3–308.8 mg/d, 4th quartile = 308.9–2,490.9 mg/d; Vitamin D: 1st quartile = 0.0–2.4 μg/d, 2nd quartile = 2.5–3.5 μg/d, 3rd quartile = 3.6–5.0 μg/d, 4th quartile = 5.1–45.8 μg/d; Zinc: 1st quartile = 0.0–11.0 mg/d, 2nd quartile = 11.1–12.6 mg/d, 3rd quartile = 12.7–14.1 mg/d, 4th quartile = 14.2–40.7 mg/d.Values in bold are statistically significant.

The hierarchical multivariate model of risk and protective factors for PACS, focusing on food group consumption (model 2) is presented in Table 5. Respectively, the higher and the intermediate consumption of white meat (HR: 0.82; 95% CI: 0.69–0.97-2nd quartile; HR: 0.81; 95% CI: 0.68–0.96-3rd quartile) and vegetables (HR: 0.82; 95% CI: 0.69–0.97-2nd quartile; HR: 0.81; 95% CI: 0.68–0.96-3rd quartile) were protective factors for PACS, whereas the higher consumption of eggs constituted an independent risk factor for the outcome (HR: 1.59; 95% CI: 1.34–1.89-4th quartile).

**Table 5:** Hierarchical multivariate model of risk and protective factors for Post-Acute COVID-19 Syndrome, highlighting food groups.

Characteristics	Post-Acute COVID-19 Syndrome		*p*-value*
	HR	95% CI	
Distal block			
Sex			
Male	1 (Ref.)	–	–
Female	**1.30**	**1.13–1.49**	**<0.001**
Age (years)			
20–29	1 (Ref.)	–	–
30–39	1.00	0.86–1.16	0.975
40–49	**1.40**	**1.17–1.68**	**<0.001**
50–74	1.17	0.94–1.46	0.153
*p* for trend	–	–	**0.002**
Family income (minimum monthly salaries)			
<4	1 (Ref.)	–	–
5–9	0.61	0.52–0.71	**<0.001**
≥10	0.51	0.44–0.59	**<0.001**
*p* for trend	–	–	**<0.001**
Intermediate block 1			
Food groups (g/d)			
White meat			
1st quartile	1 (Ref.)	–	–
2nd quartile	0.89	0.75–1.05	0.175
3rd quartile	**0.80**	**0.67–0.94**	**0.009**
4th quartile	**0.84**	**0.71–1.00**	**0.045**
*p* for trend	–	–	**0.047**
Eggs (g/d)			
1st quartile	1 (Ref.)	–	–
2nd quartile	1.01	0.85–1.20	0.883
3rd quartile	1.18	0.99–1.40	0.058
4th quartile	**1.59**	**1.34–1.89**	**<0.001**
*p* for trend	–	–	**<0.001**
Vegetables (g/d)			
1st quartile	1 (Ref.)	–	–
2nd quartile	**0.82**	**0.69–0.97**	**0.021**
3rd quartile	**0.81**	**0.68–0.96**	**0.016**
4th quartile	1.06	0.89–1.25	0.530
*p* for trend	–	–	0.130
Intermediate block 2			
Obesity			
No	1 (Ref.)	–	–
Yes	**1.48**	**1.25–1.74**	**<0.001**
Hypertriglyceridemia			
No	1 (Ref.)	–	–
Yes	**1.24**	**1.03–1.49**	**0.021**
Bronchitis			
No	1 (Ref.)	–	–
Yes	**1.28**	**1.02–1.61**	**0.030**
Proximal block			
Number of COVID-19 infections (continuous)	**1.12**	**1.02–1.23**	**0.018**
Waves of COVID-19 infection			
First	1 (Ref.)	–	–
Second	1.18	0.92–1.51	0.195
Third	**1.49**	**1.08–2.06**	**0.016**
Endemic period	1.13	0.81–1.56	0.468
Post pandemic period	1.45	0.92–2.29	0.111
Number of COVID-19 Symptoms (continuous)	**1.10**	**1.08–1.13**	**<0.001**
Searched for health care			
No	1 (Ref.)	–	–
Yes	**1.33**	**1.15–1.53**	**<0.001**
Vaccinal status*			
After COVID-19 infection	1 (Ref.)	–	–
Before COVID-19 infection	**0.68**	**0.55–0.84**	**<0.001**

CUME Study, 2016–2023.

HR = Hazard Ratio; 95% CI = 95% Confidence Interval; *p-value from Cox regression. White meat: 1st quartile = 0.0–47.5 g/d, 2nd quartile = 47.6–79.6 g/d, 3rd quartile = 79.7–116.5 g/d, 4th quartile = 116.6–1,128.1 g/d; Eggs: 1st quartile = 0.0–5.7 g/d, 2nd quartile = 5.8–12.8 g/d, 3rd quartile = 12.9–32.9 g/d, 4th quartile = 33.0–292.4 g/d; Vegetables: 1st quartile = 0.0–141.2 g/d, 2nd quartile = 141.3–195.2 g/d, 3rd quartile = 195.3–271.4 g/d, 4th quartile = 271.5–1,423.6 g/d.Values in bold are statistically significant.

## Discussion

This study employed multivariate hierarchical models to investigate independent risk and protective factors for PACS with an emphasis on COVID-19 vaccination and food consumption. Our results indicate several risk factors for PACS, including socioeconomic characteristics, pre-existing health conditions, dietary habits, COVID-19 acute period characteristics, and vaccination against COVID-19.

First, females exhibited a higher risk for PACS, consistent with previous studies, potentially due to hormonal and immunological differences (48). Estrogen exerts immunomodulatory effects, which may render women’s immune systems more susceptible to exacerbated reactions, such as the chronic inflammation observed in PACS (48, 49). Furthermore, estrogen can increase the expression of viral receptors in cells, facilitating viral entry, but it can also influence recovery and the inflammatory response post-infection (50). Additionally, the age group between 40 and 49 years was identified as a risk factor, aligning with epidemiological data indicating greater vulnerability in middle-aged adults (51).

Interestingly, higher protein and lipid intake was associated with an increased risk of PACS. While the exact relationship between dietary intake and PACS remains unclear, excessive consumption of these meats may alter the composition of the intestinal microbiota. White meats are rich in L-carnitine and choline, which serve as substrates for intestinal bacteria to produce trimethylamine (TMA), subsequently transformed into trimethylamine N-oxide (TMAO) (52, 53). TMAO can damage the vascular endothelium and activate inflammatory pathways, exacerbating systemic inflammation and predisposing individuals to the development of comorbidities that worsen COVID-19 (54, 55). Similarly, egg consumption was associated with an increased risk of PACS. While eggs are an excellent source of protein and nutrients (56, 57), excessive consumption within the context of a pro-inflammatory diet may contribute to an exacerbated inflammatory response. Further research is needed to elucidate this relationship.

Another significant factor was a prior diagnosis of obesity, strongly associated with an increased risk. This aligns with the literature highlighting obesity as a predisposing factor for severe COVID-19 and its long-term effects (58–60). Additionally, conditions like hypertriglyceridemia and bronchitis were identified as predictors of PACS, suggesting that cardiovascular and respiratory diseases may play a crucial role in the manifestation of the syndrome (61). COVID-19 directly and indirectly affects the respiratory system, and individuals with bronchitis may be more susceptible to viral infections and their sequelae due to pre-existing airway inflammation (62). This can exacerbate post-COVID recovery by worsening respiratory symptoms like shortness of breath and fatigue, as observed in our study and a multicenter cohort from Japan, South Korea, and the United Kingdom (63).

Conversely, our study identified protective factors for PACS. Higher family income emerged as a significant protective factor, aligning with longitudinal research, which showed that individuals with lower income exhibited more severe cardiovascular symptoms (64). This may reflect greater access to healthcare, better food quality, and improved living conditions, factors associated with less severe COVID-19 and its sequelae (65).

Furthermore, the intake of micronutrients like vitamin C, vitamin D, and zinc was associated with protection against PACS, suggesting that these immune response modulators may play crucial roles in reducing post-viral inflammation (66, 67).

Vitamin D exerts an essential immunomodulatory effect, balancing the immune response and reducing systemic inflammation, potentially preventing or minimizing PACS symptoms. It reduces the production of inflammatory cytokines, increases the production of antimicrobial peptides like cathelicidin, and promotes the function of regulatory T cells, which control the immune response (68). These effects help prevent chronic inflammation and exacerbated immune responses; factors frequently associated with PACS (67, 69). A population-based survey in Europe demonstrated that individuals with a blood concentration of vitamin D 25 (OH) ≥ 50 nmol/L had lower COVID-19 mortality compared to those with a blood concentration of vitamin D 25(OH) < 50 nmol/L (70).

Vitamin C and zinc modulate oxinflammation, combating reactive oxygen species and reducing the expression of TNF-α and IL-6, inflammatory cytokines associated with respiratory disease progression and PACS (71). These findings highlight the potential of nutritional adequacy as a strategy to reduce the risk of PACS and prevent complications during acute COVID-19.

When analyzing a multivariate model, higher consumption of white meat and vegetables was associated with a lower risk of PACS, likely reflecting the beneficial impact of a diet rich in lean proteins and antioxidant nutrients. White meats provide high-quality proteins and essential amino acids, crucial for immune function and tissue recovery, both important in viral infections and COVID-19 recovery (72). Vegetables are rich in vitamins and other nutrients essential for immune system function and combating oxidative stress (73–75). A nine-month prospective study in the United Kingdom, following over 3 million individuals, evaluated the association between diet quality and COVID-19 risk and severity. They observed that higher diet quality was associated with a lower risk of COVID-19 infection and milder symptoms compared to those in the lowest quartile of the diet score (76). These data reinforce the notion that a balanced diet, rich in micronutrients and high-quality proteins, can modulate the inflammatory response and improve recovery from viral diseases (72).

Another significant finding was the protective effect of vaccination against PACS. Individuals who received the vaccine before contracting the disease had a lower chance of developing PACS. This aligns with growing evidence that vaccination can reduce the risk of infection and minimize long-term disease effects (27). A meta-analysis of over 10 million patients with an average age of 50.6 years evaluated the potential protective effect of vaccines. They observed a lower prevalence of PACS symptoms in vaccinated individuals compared to unvaccinated individuals, with a decrease in symptoms (OR: 0.59; 95% CI 0.48–0.73). Furthermore, the OR of 0.77 indicated a 23% reduction in the risk of developing PACS (95% CI 0.75–0.79) associated with vaccination (28). Thus, COVID-19 vaccines play a crucial role in mitigating the severity of PACS.

### Study limitations and strengths

It is suggested that our scientific findings should be interpreted with caution due to some limitations: (1) The symptoms of PACS were self-reported. However, studies conducted with a sample with high education, such as CUME Study, indicated excellent accuracy of self-reported data (77). Furthermore, in validation studies conducted with samples from our cohort, self-reported diagnoses of different outcomes showed excellent agreement with those measured directly by physicians and other health professionals (44, 78); (2) Our sample is not representative of the Brazilian population, and limited to participants with high education. However, our participants hold high and crucial positions for the Brazilian economy, and interruption of their work activities due to illness or death may result in significant social and economic burdens for the country; (3) All participants had mild cases of COVID-19, and most did not require hospitalization or invasive procedures during treatment; (4) Exposure variables, especially socioeconomic factors, lifestyle habits and food consumption, may be modifiable over time. Therefore, this should be considered when interpreting our findings. However, in a reproducibility sub-study of our FFQ, after one year between data collections, the overall agreement of the results was excellent (92.5%), with a mean intraclass correlation coefficient of 0.76 (38); (5) Some symptoms of PACS are similar to those of perimenopause and menopause, particularly memory changes, headaches, muscle weakness, and anxiety disorders (79). Therefore, because our sample was predominantly female, with almost one-third of women over 40 years of age, there may have been difficulties in differentiating self-reported symptoms of PACS from symptoms of perimenopause and menopause.

Strengths include a robust sample size, longitudinal study design with considerable follow-up time. Additionally, this study was developed with a general target audience, not restricted to hospital discharges, broadening the understanding of the studied theme to a wider spectrum of the population. Finally, it is important to highlight that we also validate the main self-reported data used in the present study (38, 44).

## Conclusion

PACS is a high-magnitude event, constituting an important public health concern to be faced by health system managers and health professionals in the coming years after the end of the COVID-19 pandemic period.

Additionally, health policies and programs that promote a healthy diet with higher consumption of white meat, vegetables and specific micronutrients (vitamin C, vitamin D, zinc), in parallel with pre-infection COVID-19 vaccination, are essential to reduce the risk of PACS.

## Data Availability

The original contributions presented in the study are included in the article/Supplementary material, further inquiries can be directed to the corresponding author.
