# Supplementary material for: Vaccination and food consumption: association with Post-Acute COVID-19 Syndrome in Brazilian adults (CUME Study)
Source: Front Nutr. 2025 Mar 14;12:1549747. doi: 10.3389/fnut.2025.1549747 (PMC11950691; doi:10.3389/fnut.2025.1549747)
Supplement: Supplementary file 1 [file Data_Sheet_1.PDF]

Supplementary Table 1 – Food consumption characteristics of participants according to dietary reference intakes, stratifying by diagnosis of Post-Acute Covid-19 Syndrome. CUME Study, 2016-2023.

**Characteristics**	**Post-Acute COVID-19 Syndrome**					
	**No (941)**		**Yes (n = 1,124)**		**Total (n = 2,065)**	
**Food consumption**	**n**	**%**	**n**	**%**	**n**	**%**
Macronutrients (% energy)						
Carbohydrates						
Low	519	55.1	602	53.6	1,121	54.3
Adequate	408	43.4	514	45.7	922	44.6
High	14	1.5	8	0.7	22	1.1
Proteins						
Low	16	1.7	16	1.4	32	1.6
Adequate	921	97.9	1,101	98.0	2,022	97.9
High	4	0.4	7	0.6	11	0.5
Lipids						
Low	44	4.7	45	4.0	89	4.3
Adequate	361	38.3	436	38.8	797	38.6
High	536	57.0	643	57.2	1,179	57.1
Micronutrients (milligrams/day)						
Iron						
Low	18	1.9	23	2.1	41	1.9
Adequate	923	98.1	1,100	97.8	2,023	97.9
High	0	0.0	1	0.1	1	0.1
Vitamin C						
Low	86	9.1	92	8.1	178	8.6
Adequate	853	90.7	1,028	91.5	1,881	91.1
High	2	0.2	4	0.4	6	0.3
Vitamin D†						
Low	721	76.6	842	74.9	1,563	75.7
Adequate	220	23.4	282	25.1	502	24.3
High	0	0.0	0	0.0	0	0.0
Magnesium*						
Low	155	16.5	166	14.8	321	15.5
Adequate	212	22.5	307	27.3	519	25.1
High	574	61.0	651	57.9	1,225	59.3
Selenium†						
Low	27	2.9	39	3.5	66	3.2
Adequate	882	93.7	1,051	93.5	1,933	93.6
High	32	3.4	34	3.0	66	3.2
Zinc						
Low	38	4.0	45	95.9	83	4.0
Adequate	903	96.0	1,078	4.0	1,981	95.9
High	0	0.0	1	0.1	1	0.1

Note: *p-value < 0.05 by Pearson's chi-square teste; †micrograms/day.
